# Correlation of Group C Meningococcal Conjugate Vaccine Response with B- and T-Lymphocyte Activity

**DOI:** 10.1371/journal.pone.0031160

**Published:** 2012-02-08

**Authors:** James B. Wing, Lynne Smart, Ray Borrow, Jamie Findlow, Helen Findlow, Andrew Lees, Robert C. Read, Andrew W. Heath

**Affiliations:** 1 Department of Infection and Immunity, University of Sheffield Medical School, Sheffield, United Kingdom; 2 Vaccine Evaluation Unit, Health Protection Agency, Manchester Royal Infirmary, Manchester, United Kingdom; 3 Inflammation Sciences Research Group, University of Manchester, Manchester, United Kingdom; 4 Fina Biosolutions LLC, Rockville, Maryland, United States of America; Tulane University, United States of America

## Abstract

Despite the success of conjugate vaccination against meningococcal group C (MenC) disease, post-vaccination, some individuals still exhibit rapid waning of initially protective bactericidal antibody levels. The mechanism of this relative loss of humoral protection remains undetermined. In this report we have investigated the relationship between T- and B-cell activation and co-stimulation and the loss of protective antibody titers. We have found that healthy volunteers who lose protective MenC antibody levels one year after receipt of glycoconjugate vaccine exhibit no detectable cellular defect in polyclonal B- or T-cell activation, proliferation or the B-memory pool. This suggests that the processes underlying the more rapid loss of antibody levels are independent of defects in either initial T- or B-cell activation.

## Introduction

Since the introduction of meningococcal group C conjugate (MCC) vaccines, rates of infection have fallen significantly in the UK [1], [2]. However a number of cases of secondary vaccine failure, in which an initially protective level of antibody wanes over time leading to vulnerability to infection, still occur [3].

While plain polysaccharide vaccines are able to induce protective antibody responses via direct stimulation of B-cells by cross-linking of the B-cell receptor, this stimulus alone does not induce large scale switching of B-cells to long lived plasma cells or memory B-cells. For longer lasting immunity, T-cell help is required [4], [5]. In MCC vaccination, peptide (usually CRM_197_ or tetanus toxoid) conjugated to polysaccharide is presented to T-cells via MHC-II on B-cells or other antigen presenting cells along with co-stimulatory signalling via CD80/86. This triggers T-cell help and the delivery of cognate signals such as CD40-CD40 ligand interaction with polysaccharide specific B-cells [6] leading to differentiation into both antibody producing plasma cells and B-memory cells [7]. This memory B-cell population is vital for both immunological memory and longer term antibody production as memory B-cells are able to renew the population of shorter lived plasma cells over time [8]. As a result, successful and long lasting responses to MCC vaccination requires both B-cell recognition of polysaccharide and effective delivery of T-cell help to those B cells.

We have previously found that waning of protective antibody levels occurs in a small but significant proportion of subjects vaccinated with MCC vaccine after one year [9]. To determine whether the loss of putative protective antibody levels in this population is related to intrinsic host peripheral blood B- and T-lymphocyte activity, we have used an *in vitro* cellular assay that models the cognate interactions between B and T-cells that occur following conjugate vaccination. We measured the response of B-cells to a polyclonal mimic of bacterial capsular polysaccharide, delivery of T-cell help to B-cells, and T-cell responses themselves. Using this model we have previously found polyclonal B and T cell defects in both unvaccinated [10] and previously MCC vaccinated [11] patients who have suffered meningococcal disease and in patients who have suffered an episode of invasive pneumococcal disease [12] suggesting that defects in B and T cell function may play a common role in susceptibility to infection by encapsulated bacteria.

In this case we found no evidence of a definable defect in either polyclonal B-or T-cell activation, proliferation or the existing B-memory pool, in individuals who have lost protective antibody levels one year post vaccination. This suggests that the processes underlying the more rapid loss of protective antibody levels are independent from defects in initial T- and B-cell activation.

## Methods

### Study population and clinical procedures

116 undergraduate and postgraduate students (median age 23, range 19 to 39 years) presenting for meningococcal vaccination were vaccinated with meningococcal group C polysaccharide conjugated to tetanus toxoid (NeisVac-C, Baxter). Antibody responses were measured in serum taken at 0 and 28 days, and again one year later, as reported previously [9]. In addition a 50 ml blood sample was also taken for analysis of cellular activation as described below.

Of the original 116 subjects, 89 returned 1 year later. As described previously [9] all subjects initially responded to vaccination but 11 subjects were found to have serum bactericidal antibody (SBA) levels that had dropped below the threshold of protection, with rabbit complement derived SBA titers r below 8 (SBA<8) [13], [14].

This SBA low group was then compared to age and sex matched controls who had retained protective SBA levels (SBA high group) using the cell stimulation assay described below. Since there is still some uncertainty as to the protected status of individuals with intermediate SBA titers, equal to or above 8 but below 128 [14], [15] the SBA high group was selected only from individuals with SBA titers equal to or above 128.

All subjects gave written informed consent and the procedures were approved by the Central Office for Research Ethics (United Kingdom, Ref. 04/S0501/34).

### Cell stimulation materials

B-cells were stimulated with the TI-II antigen mimic α-∂-dextran (α-∂-dex). α-∂-dex consists of multivalent anti-IgD antibodies conjugated to dextran and has been used in a number of studies as a polyclonal B-cell activator [10], [11], [16], [17], [18]. α-∂-dex was made as described previously [19], [20]. α-∂-dex polyclonally activates B-cells by cross-linking IgD within the B-cell receptor complex (BCR).

T-cells were activated by plate-bound anti-CD3 either alone or with anti-CD28 (both Caltag laboratories). In this system anti-CD3 mimics T-cell receptor signalling while anti-CD28 mimics the CD80/86 co-stimulation of T-cell CD28.

### Cell isolation and stimulation

Peripheral blood mononuclear cells (PBMCs) were isolated from fresh heparinised blood by centrifugation with lymphocyte separation media (Biowhittaker) and removal of the buffy coat before washing. For proliferation experiments cells were also stained with 2 µM CFSE (Invitrogen). Cells were enumerated by haemocytometer with dead cells excluded on the basis of trypan blue (Sigma) staining. Cells were added to 48-well plates at a concentration of 2×10^6^ cells per ml in a final volume of 500 µl RPMI 1640 plus L-glutamine (Gibco) containing 10% autologous plasma.

For analysis of basal CD3 and CD28 expression, and memory B-cell populations, cells were harvested at day 0.

For analysis of B- and T-cell responses to stimulation, T-cells were stimulated with 0.1 µg/ml plate-bound anti-CD3 or 0.5 µg/ml plate-bound anti-CD3 and anti-CD28. B-cell stimulation was provided by 1 µg/ml α-∂-dex both in the presence and absence of the T-cell stimulators. Except where otherwise noted, cells were incubated at 37°C, 5% CO_2_ in a humidified atmosphere for 96 hours before harvesting; supernatants were also collected for cytokine analysis.

### Antibodies and cell staining

For immunofluorescence staining, the following antibody/cell stains were used in the following combinations. Proliferation and activation of B- and T-cells was measured by multi-parameter flow cytometry with the following combination of fluorochromes; Day 0 analysis- IgD PE, CD3 Alexa700, CD4 APC, CD19PE-Cy7, CD28 Alexa488, CD27 APC-Alexa750 (All Caltag Laboratories). Activation analysis- CFSE (Invitrogen), CD14 Pacific Blue, CD25 PE, CD86 PE-Alexa700, CD19 PE-Cy7, CD4 APC, CD27 APC-Alexa750 (all Caltag Laboratories) and UV Live/Dead (Invitrogen). Immunofluorescence staining for flow cytometric analysis was performed by incubating 1×10^6^ PBMCs with the relevant antibodies suspended in PBS with 0.1% BSA for 1 hour at 4°C before washing and fixation with 1% paraformaldehyde.

### Flow cytometry

Cells were analysed using a LSRII flow cytometer (BD). T- and B-cells were identified by a sequential gating strategy in the following order; Forward versus side scatter (FSC/SSC) gating on lymphocytes, Doublet discrimination (FSC-Area vs. FSC-Height), live cell discrimination by UV Live/Dead dye, monocyte exclusion by gating on CD14 negative cells and finally gating on the relevant cell populations (CD4, CD19). A total of 100,000 events were collected for each analysis. Compensation was done post-hoc with the compensation package of FlowJo™ software (Tree Star) in conjunction with anti-mouse compensation beads (BD) or cell based staining in the cases of CFSE and UV Live/Dead. In order to ensure reproducibility, cytometer setup and tracking was performed using Cytometer setup and tracking beads (BD) and FACSdiva™ software (BD).

CFSE proliferation was measured by the use of the FlowJo proliferation analysis platform to calculate the divisional index (Mean number of divisions undertaken by each cell). Activation marker levels were assessed by median fluorescence intensity (MFI) of the relevant conjugated fluorochromes.

### Cytokine measurement

Measurement of cytokines (IL-5, -10, -13, 17, TNF-α, IFN-γ and IL-1β) in collected supernatants was carried out using Cytometric Bead Array Flexsets (BD) according to manufacturers' instructions. Results were collected on a BD FACSArray using BD FACSArray software for acquisition and FCAP array software (Soft Flow) for analysis.

### Statistics

Distribution of results were analysed graphically and by the D'Agostino-Pearson test to establish if results had a parametric distribution. The majority of the cellular activation and proliferation data was found to be normally distributed once transformed by square rooting. Where data were normally distributed one-way ANOVA with post-tests were used. In order to reduce the potential for type-II errors induced by multiplicity corrections we restricted statistical analysis to relevant comparisons by use of the Bonferroni selected pairs post-test.

## Results

### Individuals who have lost protective SBA titer one year post-vaccination do not have altered T-cell marker or memory B-cell distribution

**Figure 1:**
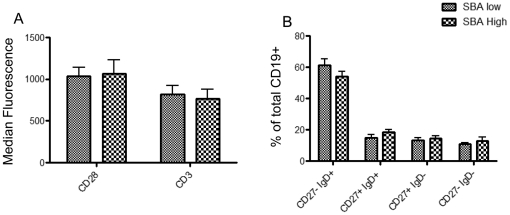
T-cell CD3 and CD28 expression and B-cell memory pool distribution. PBMCs were extracted from subjects with a MenC SBA titer <8 and matched controls one year post vaccination and levels of T-cell CD3 and CD28 expression B-cell memory subsets were examined by flowcytometry. n = 11, One-way ANOVA+ Bonferroni selected pairs post-test (SBA low vs. SBA high).

To determine if any observed differences in anti-CD3 and anti-CD28 response were due to differences in CD3 and CD28 expression by CD4^+^ cells we analysed the level of surface expression of these markers. No significant difference was seen between the SBA low group (SBA titer <8) and the matched controls (**Figure 1a**).

In addition we analysed the relative ratios of memory B-cell subsets (specifically CD27+ IgD+ memory cells , CD27+ IgD− isotype-switched memory cells, CD27− IgD+ naive B-cells and the CD27− IgD− population comprising of a mixture of CD27− switched memory cells and exhausted memory cells[21], [22], [23]) to investigate the possibility that a loss of protective antibody might be due to a pre-existing defect in memory B-cell generation or isotype switching. Also since we used anti-IgD based stimulation it was necessary to ensure that there was no difference in the numbers of IgD expressing B-cells. No significant difference was found between the low group and SBA high controls (**Figure 1b**).

### Individuals who have lost protective SBA titers one year post-vaccination do not have a T- or B- cell activation defect

**Figure 2:**
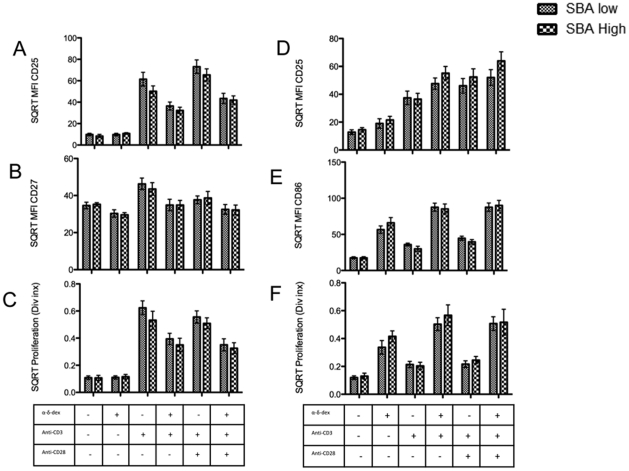
T-cell and B-cell activation/proliferation in response to polyclonal stimuli. PBMCs were extracted from subjects with a MenC SBA titer <8 and from matched controls one year post vaccination and stimulated for 96 hours with plate bound αCD3±αCD28 and/or the TI-II antigen mimic, α-δ-dex. Activation and proliferation was measured by expression of A) CD25 Median Fluorescence Intensity (MFI) of CD4^+^ cells, B) CD27 MFI of CD4^+^ cells, C) Proliferation of CD4^+^ cells, D) CD25 MFI CD19^+^ cells, E) CD86 MFI of CD19^+^ cells and F) Proliferation of CD19^+^ cells. n = 11, data square rooted for normality, One-way ANOVA+ Bonferroni selected pairs post-test (SBA low vs. SBA high).

To investigate the possibility that poor retention of anti-MenC SBA concentrations may be related to an observable defect in cellular activation of B- and T-cells we compared the response to polyclonal stimulation of SBA low donors (at 12 months) to age and sex matched controls with protective SBA titers. There was a trend towards increased T-cell activation/proliferation with concurrent lower B-cell activation seen in the SBA low donors in comparison to the SBA high donors but this was not statistically significant (**Figure 2**). In addition to analysis of the total B-cell population we also examined any differences in the CD27^+^ memory population alone, again we did not see any significant differences between the SBA low and high groups, in B-cell proliferation or activation marker expression (**Figure S1**).

### Individuals who have lost protective SBA titers one year post-vaccination do not have a detectable change in ex-vivo cytokine production by polyclonally stimulated lymphocytes

**Figure 3:**
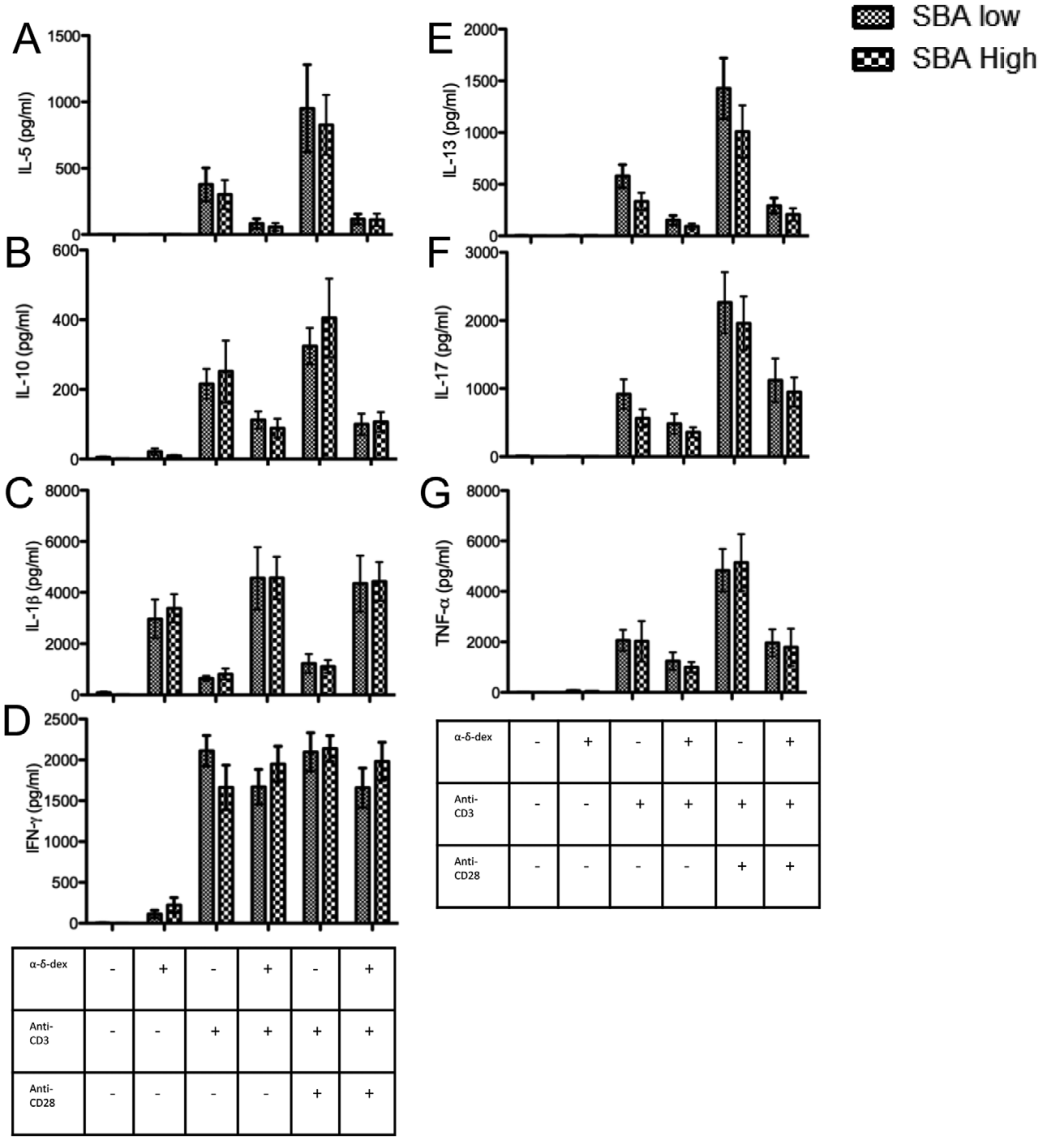
Cytokine production in response to polyclonal stimuli. PBMCs were extracted from subjects with an MenC SBA titer <8 one year post vaccination and matched controls and stimulated for 96 hours with plate bound αCD3±αCD28 and/or the TI-II antigen mimic, α-δ-dex. Supernatants were collected and cytokine concentrations assessed by cytometric bead array. n = 10. One-way ANOVA+ Bonferroni selected pairs post-test (SBA low vs. SBA high).

There was no difference between the SBA low and high groups in the levels of induced cytokines found in the supernatants after 96 hours of stimulation (**Figure 3**).

## Discussion

We did not see a definable difference in the cellular measurements assessed in this study between the group of donors who lost protective MenC SBA levels one year post vaccination and those who did not. This adds further information to our previous findings that patients who have suffered meningococcal disease despite vaccination have a definable polyclonal T-cell defect both in T-cell activation and delivery of T-cell help to B-cells [11]. The previous study investigated vaccinees who actually succumbed to disease, rather than those who lost immunological protection defined by an immunological surrogate of protection, and are therefore a distinct population to the normal volunteers studied here. As described by Goldschneider and colleagues the majority of individuals without a protective SBA level colonised by meningococci do not develop disease [24]. Thus, an SBA titer below the protective cut off is necessary, but may not be sufficient, for susceptibility to disease. Taken together with our previous data in which we identified a defect in T cell polyclonal activation in vaccine failures who had succumbed to disease [11], our results would support this hypothesis. Were the vaccine failures (who succumbed to disease) from the same population as our SBA low group (at one year), then the same T cell defect should have been evident. In the previous study [11] we concluded that the observed deficiency in T-cell help to B-cells may have been the cause of the loss of SBA levels due to poor induction of long lasting B-cell immunity, however in the light of the results presented here it now seems more likely that the observed deficiency is an additional risk factor that determines susceptibility to infection in individuals who have lost protective SBA levels, rather than the cause of the loss of SBA itself.

In mice at least, the response to whole polysaccharide on whole encapsulated bacteria is more close to a T-dependent response than to a T-independent response to polysaccharide alone [25]. It is possible then that susceptibility to disease depends both on a level of circulating SBA below the threshold, and on a poor, or slow (T-dependent) response to whole organisms on colonisation or perhaps more likely, on invasion.

Since the half-life of antibody producing plasma cells is estimated to be around 40 days [8] it seems likely that any loss of antibody protection may be due to poor initial induction of B-memory cells. However, we did not find any evidence of difference in either the starting B-memory pool or in of the CD27+ memory B-cell response to polyclonal stimulation, suggesting that any loss of antibody protection is not due to an intrinsic imbalance in memory B-cell production, class switching or an inability of the cells to respond to stimulation. This may be supported by the finding that the number and persistence of Men-C specific memory B-cells induced by MCC vaccination is not age dependent [26]. Given that the protection offered by MCC vaccination does vary with age [27] it then seems likely that variation in memory B-cell populations may not be the critical factor in determining vaccine efficacy.

As with cellular activation, cytokine levels were not significantly different between the SBA low and high groups demonstrating no difference in the Th1, Th2 or Th17 cytokine profiles.

A number of caveats must be attached to these data. First we are reliant on peripheral blood lymphocytes due to the difficulties in accessing other immunological compartments in humans. However, it has been suggested that memory B-cells found in peripheral blood are representative of the total cell population [28]. The use of polyclonal stimulation by α-δ-dextran and anti-CD3 is a broad method necessitated by the great difficulty in detecting changes in the extremely small proportion of circulating B-or T-cells that would respond to stimulation by a single antigen, however this methodology has been previously used to successfully identify immune-defects in several disease contexts [11], [12], [16]. While we do not rule out the possibility of a more antigen specific defect being present we are able to conclude that the groups shown here lack the previously described polyclonal activation defects that we have identified in several cohorts of meningococcal disease sufferers [10], [11].

The findings presented here suggest that the observed waning of antibody levels seen in the normal population is not due to any detectable defect in initial T or B-cell activation. We suggest that the defects in T-cell activation observed amongst patients who have experienced disease are an additional risk factor in antibody-unprotected groups rather than the cause of the loss of antibody.

## Supporting Information

Figure S1 **Memory and non-memory B-cell activation/proliferation in response to polyclonal stimuli.** PBMCs were extracted from subjects with a MenC SBA titer <8 and from matched controls one year post vaccination and stimulated for 96 hours with plate bound αCD3±αCD28 and/or the TI-II antigen mimic, α-δ-dex. Activation and proliferation was measured by expression of A&D) CD25 MFI of CD19^+^ cells, B&E) CD86 MFI of CD19^+^ cells and C&F) Proliferation of CD19^+^ cells. A, B and C) CD27^+^ memory B-cells. D, E and F) CD27^−^ non-memory B-cells. n = 11, data square rooted for normality, One-way ANOVA+ Bonferroni selected pairs post-test (SBA low vs. SBA high).
(TIF)
