# Correction: Correlation of Group C Meningococcal Conjugate Vaccine Response with B- and T-Lymphocyte Activity

**DOI:** 10.1371/annotation/45ca0028-74a1-427d-ba95-3dbd2acc6b80

**Published:** 2012-11-14

**Authors:** James B. Wing, Lynne Smart, Ray Borrow, Jamie Findlow, Helen Findlow, Andrew Lees, Robert C. Read, Andrew W. Heath

Rachel A. Foster and Jennifer Carlring were erroneously omitted from the author byline. The correct author list with affiliations is:

James B. Wing^1^, Lynne Smart^1^, Ray Borrow^2,3^, Jamie Findlow^2,3^, Helen Findlow^2^, Andrew Lees^4^, Rachel A. Foster^1^, Jennifer Carlring^1^, Robert C. Read^1^*, Andrew W. Heath^1^

Their contributions are:

Helped design and troubleshoot the assay: RAF, JC

Helped with the recruitment model: RAF 

