# Supplementary figures and images for: Correlation of Group C Meningococcal Conjugate Vaccine Response with B- and T-Lymphocyte Activity
Source: PLoS One. 2012 Feb 8;7(2):e31160. doi: 10.1371/journal.pone.0031160 (PMC3275607; doi:10.1371/journal.pone.0031160)

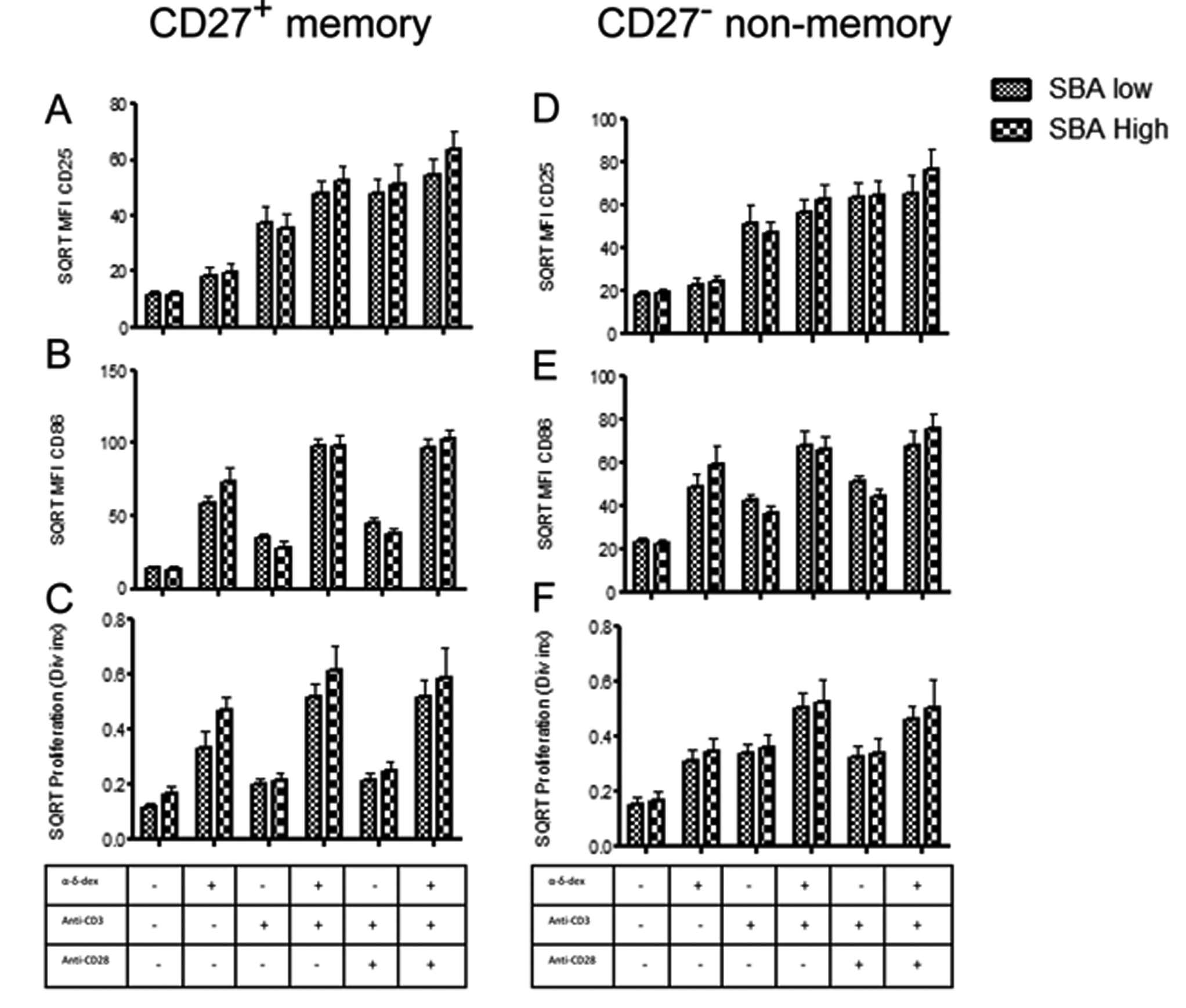

Supplement: Figure S1 — Memory and non-memory B-cell activation/proliferation in response to polyclonal stimuli. PBMCs were extracted from subjects with a MenC SBA titer <8 and from matched controls one year post vaccination and stimulated for 96 hours with plate bound αCD3±αCD28 and/or the TI-II antigen mimic, α-δ-dex. Activation and proliferation was measured by expression of A&D) CD25 MFI of CD19+ cells, B&E) CD86 MFI of CD19+ cells and C&F) Proliferation of CD19+ cells. A, B and C) CD27+ memory B-cells. D, E and F) CD27− non-memory B-cells. n = 11, data square rooted for normality, One-way ANOVA+ Bonferroni selected pairs post-test (SBA low vs. SBA high). (TIF) [file pone.0031160.s001.tif]
